# Dynamics of Protein Accumulation from the 3′ End of Viral RNA Are Different from Those in the Rest of the Genome in Potato Virus A Infection

**DOI:** 10.1128/JVI.00721-19

**Published:** 2019-09-12

**Authors:** Shreya Saha, Anders Hafren, Kristiina Mäkinen

**Affiliations:** a Faculty of Agriculture and Forestry, Department of Microbiology, Viikki Plant Sciences Center, University of Helsinki, Helsinki, Finland; University of Maryland, College Park

**Keywords:** helper component proteinase, HCPro, polyprotein, potyvirus, ribosomal protein P0, stoichiometry of protein production, translation, genome-linked viral protein, VPg

## Abstract

One large open reading frame (ORF) encodes 10 potyviral proteins. We compared the accumulation of cylindrical inclusion (CI) protein from the middle, coat protein (CP) from the 3′end, and *Renilla* luciferase (RLUC) from two distinct locations in potato virus A (PVA) RNA. 5′ RLUC was expressed from an *rluc* gene inserted between the P1 and helper component proteinase (HCPro) cistrons, and 3′ RLUC was expressed from the gene inserted between the RNA polymerase and CP cistrons. Viral protein and RNA accumulation were quantitated (i) when expressed from PVA RNA in the presence of ectopically expressed genome-linked viral protein (VPg) and auxiliary proteins and (ii) at different time points during natural infection. The rate and timing of 3′ RLUC and CP accumulation were found to be different from those of 5′ RLUC and CI. Ectopic expression of VPg boosted PVA RNA, 3′ RLUC, and, together with HCPro, CP accumulation, whereas 5′ RLUC and CI accumulation remained unaffected regardless of the increased viral RNA amount. In natural infection, the rate of the noteworthy minute early accumulation of 3′ RLUC accelerated toward the end of infection. 5′ RLUC accumulation, which was already pronounced at 2 days postinfection, increased moderately and stabilized to a constant level by day 5, whereas PVA RNA and CP levels continued to increase throughout the infection. We propose that these observations connect with the mechanisms by which potyvirus infection limits CP accumulation during early infection and specifically supports its accumulation late in infection, but follow-up studies are required to understand the mechanism of how this occurs.

**IMPORTANCE** The results of this study suggest that the dynamics of potyviral protein accumulation are regulated differentially from the 3′ end of viral RNA than from the rest of the genome, the significance of which would be to satisfy the needs of replication early and particle assembly late in infection.

## Abstract (precis)

The results of this study suggest that the dynamics of potyviral protein accumulation are regulated differentially from the 3′ end of viral RNA than from the rest of the genome, the significance of which would be to satisfy the needs of replication early and particle assembly late in infection.

## INTRODUCTION

Potyviruses encapsidate a positive-sense single-stranded RNA genome which is approximately 10 kb in size and carries a genome-linked viral protein (VPg) at its 5′ end and a poly(A) tail at its 3′ end. Potyviral RNA consists of one large open reading frame (ORF) encoding a polyprotein that can be cleaved into 10 functional proteins by virus-encoded proteinases (reviewed in reference 1). It was a long-standing belief that all potyviral proteins are produced in equimolar amounts as part of the polyprotein. This paradigm changed by the finding of a small ORF called “pretty interesting *Potyviridae* ORF” (PIPO) (2). PIPO overlaps the P3 cistron and is expressed in fusion with the N-terminal region of P3 (P3N-PIPO) via a frameshift originating from transcriptional slippage (3, 4). This strategy results in the production of one long polyprotein extending from the P1 protein to the coat protein (CP) as the major protein and one short one from P1 to PIPO as the minor product; consequently, P1 and helper component proteinase (HCPro) are produced in larger quantities and P3N-PIPO is produced in smaller quantities than the other potyviral proteins.

Plant viral CPs have a major role in viral encapsidation and movement, but they also regulate viral gene expression (reviewed in reference 5). The need for CP is usually minimal during active viral gene expression in the early stages of infection compared to its high demand at the time of encapsidation. It is an intriguing question of how the CP amounts are regulated in the course of potyvirus infection. The stability of CP of plum pox virus (PPV) (genus *Potyvirus*) is regulated by concurrently occurring phosphorylation and *O*-GlcNAcylation at its N terminus (6). Our previous studies revealed that accumulation of potato virus A (PVA) (genus *Potyvirus*) CP is under tight control. Phosphorylation of CP by protein kinase CK2 (7) and a functional heat shock protein HSP70/HSP40 host chaperone pathway targeting CP for proteasomal degradation (8) are both essential for PVA replication (9). We proposed previously (9) that a transient translational block formed by binding of the nonphosphorylated CP on viral RNA (8–10) allows time for replication complex formation prior to its removal from viral RNA by the chaperones. While CP modifications and the host chaperones are pivotal in controlling potyviral CP functions during viral gene expression, the question remains as to how high enough CP accumulation can be achieved to fulfill the needs of encapsidation. We have proposed that CP accumulation eventually overpowers the HSP70/HSP40 regulative capacity, which leads to ceased translation and replication (5, 11). It can be hypothesized that shutdown of the degradation pathway contributes to CP accumulation for particle formation. HCPro of PPV was found to stabilize PPV CP and to be important for particle encapsidation (12, 13). While the abundant presence of HCPro toward the end of infection likely promotes CP accumulation and encapsidation, it is possible that yet another mechanism exists to boost specifically potyviral CP production. As proposed previously (14), one potential mechanism to achieve this is a VPg-mediated translational mechanism that we have described for PVA (14–16).

VPg has multiple functions during potyvirus infection, for example, in replication and long-distance movement (reviewed in reference 1). An interaction between potyviral VPg and host eIF4E or eIF(iso)4E is required for successful potyvirus infection (reviewed in references 17–19). Among the important VPg functions are those associated with viral RNA translation. While the potyviral 5′ untranslated region (UTR) confers cap-independent translation by serving as an internal ribosome entry site (IRES) (20, 21), the addition of VPg moderately enhances cap-independent translation from the potyviral 5′ UTR *in vitro* (22). When expressed ectopically *in vivo*, it increases PVA RNA accumulation and viral translation substantially in a VPg concentration-dependent manner (14). While VPg is the key component of this regulatory mechanism, other essential coregulators are the PVA 5′ UTR and HCPro as well as the host proteins eIF(iso)4E, ribosomal stalk protein P0, and VARICOSE (VCS) (14–16). In the present study, we found that ectopic expression of VPg associated with enhanced PVA RNA accumulation is reflected as enhanced expression of *Renilla* luciferase (RLUC) from the 3′ end of PVA RNA (3′ RLUC), whereas expression of *Renilla* luciferase from the 5′ end (5′ RLUC) surprisingly remains unaffected. Together with the finding that the expression of the 3′-end-encoded proteins 3′ RLUC and CP continues to increase until the later stages of infection, whereas the 5′-end-encoded 5′ RLUC level stabilizes in the course of infection, our hypothesis is that a mechanism that provides sufficient amounts of replication proteins early and CP late in virus infection may exist.

## RESULTS

**FIG 1:**
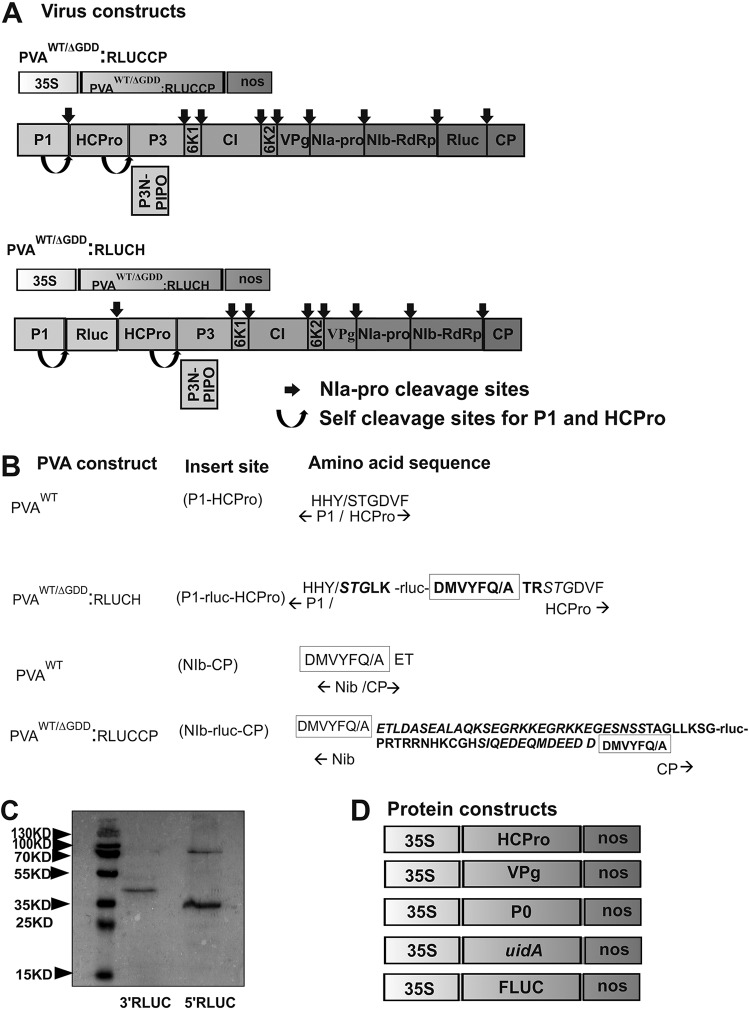
Schematic representation of the constructs. (A) Wild-type (WT) and replication-deficient (ΔGDD) PVA constructs. The PVA^WT/ΔGDD^:RLUCCP construct allows expression of 3′ RLUC from a location between the NIb and CP cistrons in PVA RNA. PVA^WT/ΔGDD^:RLUCH allows expression of 5′ RLUC from a location between the P1 and HCPro cistrons in PVA RNA. (B) The extra amino acid sequences surrounding 5′ RLUC and 3′ RLUC. PVA NIa-Pro proteinase recognition sites are marked with a box. P1 and NIa cleavage sites are denoted with a slash. Engineered extra RLUC-attached amino acids are in boldface type. Duplicated amino acids from the PVA genome in P1/HCPro and NIb/CP are marked in italics. (C) Efficiency of RLUC processing from PVA polyprotein. Samples were collected from PVA^WT^:RLUCCP- and PVA^WT^:RLUCH-infected N. benthamiana plants at 3 dpi and subjected to Western blot analysis with anti-RLUC antibodies. (D) Protein expression constructs were cloned under the control of the 35S promoter and contained a *nos* terminator.

We tagged an infectious cDNA (icDNA) clone of PVA with the *Renilla* luciferase gene (*rluc*) for accurate quantitation of PVA gene expression (23). Previously, we found that expression of the *rluc* gene located between the NIb and CP cistrons at the 3′ side of PVA genomic RNA becomes enhanced in the presence of ectopically expressed VPg and even more so together by VPg and its coregulator ribosomal protein P0 (14, 15). In the present study, we set to compare VPg-enhanced translation with two different RLUC-expressing wild-type (WT) PVA constructs: PVA^WT^:RLUCCP, the very same construct as described previously (14, 15), and PVA^WT^:RLUCH, having *rluc* located between the P1 and HCPro cistrons at the 5′ side of the viral genome. The PVA^WT^:RLUCH construct was described previously (24), under the name pOLO. These constructs are schematically presented in Fig. 1A. Along with the wild-type viral RNAs, their replication-deficient versions having a deletion in the RNA polymerase NIb active site (ΔGDD), called PVA^ΔGDD^:RLUCCP and PVA^ΔGDD^:RLUCH, were also used in our assays. As can be appreciated from Fig. 1A, all PVA proteins retain their natural amino acid sequence upon proteolytic processing of the viral polyprotein.

RLUC expressed from the PVA^WT^:RLUCH construct (5′ RLUC) is surrounded by an HHY/S cleavage site recognized by P1 proteinase and DMVYFQ/A recognized by NIa-Pro, and RLUC expressed from PVA^WT^:RLUCCP (3′ RLUC) is surrounded by two DMVYFQ/A NIa-Pro cleavage sites (Fig. 1B). Western blot analysis of PVA^WT^:RLUCH-infected Nicotiana benthamiana leaves revealed that most of the 37-kDa 5′ RLUC was processed apart from the viral proteins (Fig. 1C). The resulting 5′ RLUC carries an extra 5 amino acids at its N terminus and 6 at its C terminus. The only product observed in the PVA^WT^:RLUCCP-infected leaves by Western blot analysis was the fully processed 3′ RLUC (Fig. 1C). Due to the cloning strategy, which was described in detail previously (7), the resulting 3′ RLUC, approximately 41 kDa in size, carries an extra 27 amino acids of CP sequence at its N terminus and 18 C-terminal amino acids of NIb sequence at its C terminus. Since the amino acid sequences at the N and C termini of 5′ RLUC and 3′ RLUC are not identical, and these two proteins are produced from two different PVA constructs, their RLUC activity values are not compared to each other. Therefore, in all of the experiments described below, we monitor changes in the activities of a particular RLUC version one at a time. The VPg, HCPro, and P0 expression constructs are schematically depicted in Fig. 1D.

### Ectopically expressed VPg enhances 3′ RLUC but does not enhance 5′ RLUC expression.

**FIG 2:**
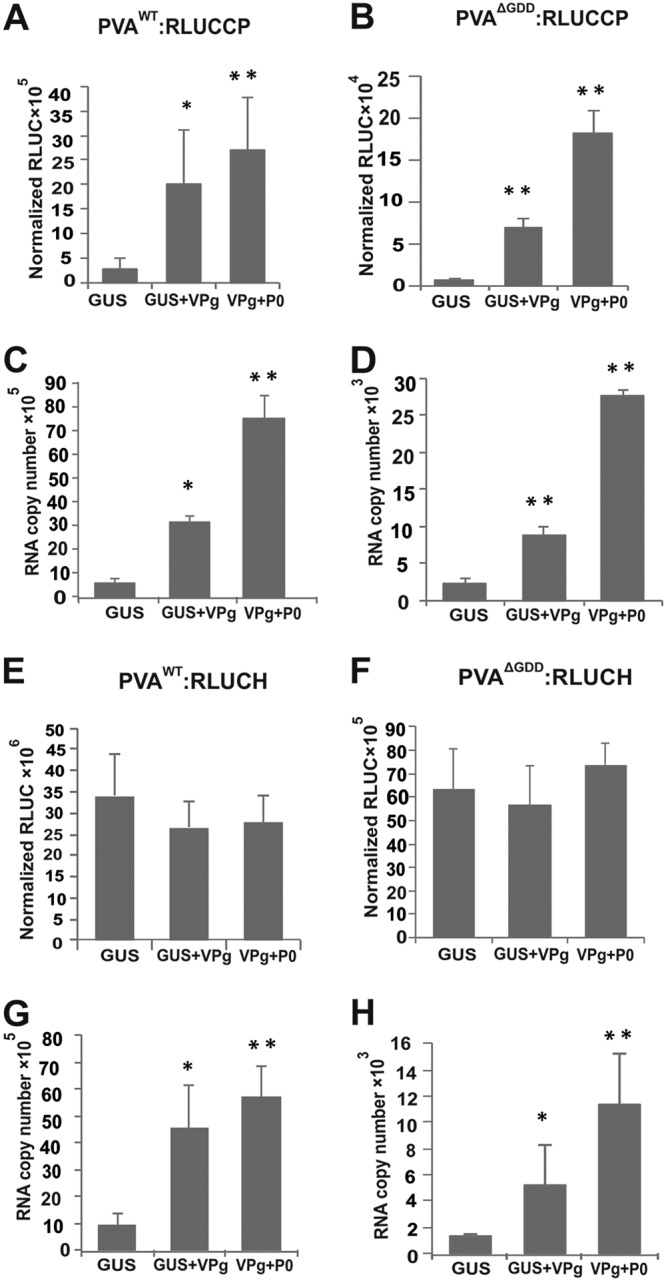
VPg stabilizes PVA RNA and selectively enhances 3′ RLUC expression. (A to D) Normalized 3′ RLUC activity from PVA^WT/ΔGDD^:RLUCCP (A and B) and PVA^WT/ΔGDD^:RLUCCP RNA accumulation (C and D) were determined in the presence of ectopically expressed GUS, VPg, and VPg+P0 at 3 dpi. (E to H) Normalized 5′ RLUC activity from PVA^WT/ΔGDD^:RLUCH (E and F) and PVA^WT/ΔGDD^:RLUCH RNA accumulation (G and H) were determined in the presence of ectopically expressed GUS, VPg, and VPg+P0 at 3 dpi. Experiments were performed at least in triplicate. Data represent results from one representative experiment, and the bars display means (*n* = 3 or more) ± SD. Student’s *t* test shows which of the samples are significantly different compared to the GUS control (*, *P* < 0.05; **, *P* < 0.01).

First, we asked whether overexpression of VPg and VPg with P0 (VPg+P0) can upregulate 5′ RLUC activities from PVA^WT^:RLUCH and PVA^ΔGDD^:RLUCH similarly as previously detected for 3′ RLUC from PVA^WT^:RLUCCP and PVA^ΔGDD^:RLUCCP (14, 15). We *Agrobacterium* infiltrated beta-glucuronidase (GUS), VPg, and VPg+P0 into Nicotiana benthamiana plants together with either PVA^WT^:RLUCCP, PVA^ΔGDD^:RLUCCP, PVA^WT^:RLUCH, or PVA^ΔGDD^:RLUCH and quantitated viral gene expression in terms of 5′ RLUC and 3′ RLUC activities at 3 days postinfection (dpi). The result shows that ectopically expressed VPg and VPg+P0 increased 3′ RLUC expression from both PVA^WT^:RLUCCP and PVA^ΔGDD^:RLUCCP significantly over the GUS control (Fig. 2A and B). This is in line with our previous reports (14, 15). Unlike 3′ RLUC, no increase in 5′ RLUC activity over the GUS control was detected in the presence of ectopically expressed VPg and VPg+P0 from either PVA^WT^:RLUCH or PVA^ΔGDD^:RLUCH (Fig. 2E and F). This result suggests that ectopically expressed VPg affects the accumulation of 3′ RLUC differently than it affects that of 5′ RLUC.

PVA RNA amounts and 3′ RLUC expression are upregulated proportionally by VPg (14, 15). We hypothesized that PVA RNA accumulation should be increased similarly by VPg regardless of the position of the *rluc* cistron in the viral genome. To test this, we subjected the samples described above to reverse transcription-quantitative PCR (qRT-PCR). The results showed significantly higher PVA RNA accumulation from all of the constructs in the presence of VPg and VPg+P0 than in the presence of GUS (Fig. 2C, D, G, and H). This result suggests that the position of *rluc* in viral RNA does not influence the VPg-mediated enhancement of PVA RNA accumulation. Importantly, this excludes the possibility that the presence of the *rluc* cistron at the 3′ end of PVA RNA would cause the enhanced RNA accumulation. Since both PVA^WT^ and PVA^ΔGDD^ RNAs accumulated at higher levels, it is likely that this is due to VPg-mediated protection of viral RNA from degradation and not due to VPg-mediated enhancement of replication. Most intriguingly, VPg enhanced PVA:RLUCH RNA accumulation without increasing its 5′ RLUC production.

### Western blot analyses provide further support for VPg-mediated enhancement of 3′-end PVA RNA translation.

**FIG 3:**
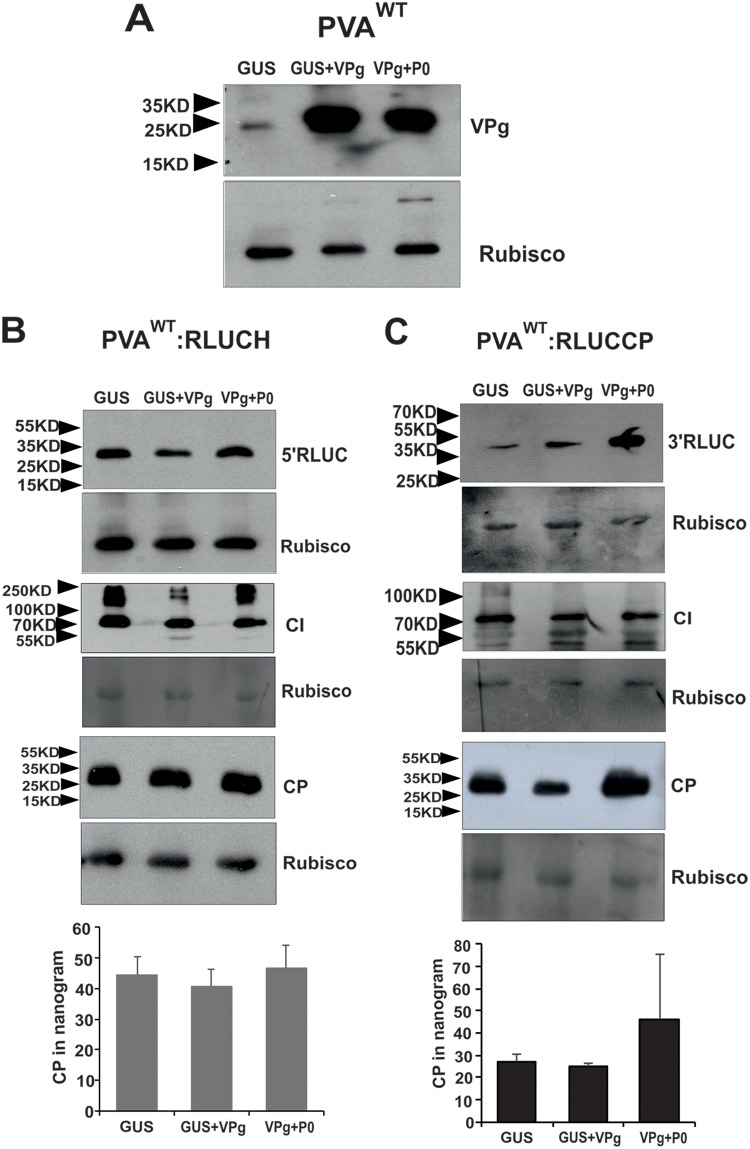
Contrary to 5′ RLUC, CI, and CP, an increased amount of 3′ RLUC accumulates in response to the ectopic expression of VPg and P0. Nicotiana benthamiana plants were *Agrobacterium* infiltrated with either PVA^WT^:RLUCH or PVA^WT^:RLUCP along with the GUS, VPg, or VPg+P0 expression construct, as indicated. (A) Verification of VPg overexpression in virus-infected plants. (B and C) Western blot analyses of 5′ RLUC, 3′ RLUC, CI, and CP and ELISAs to quantitate CP amounts from PVA^WT^:RLUCH (B)- and PVA^WT^:RLUCCP (C)-infected plants. Rubisco was used as the loading control. The positions of molecular mass markers are shown on the left. Experiments were performed at least in triplicate. Data represent results from one representative experiment, and the bars display means (*n* = 3 or more) ± SD. Student’s *t* test revealed no significant changes between the ELISA samples.

**FIG 4:**
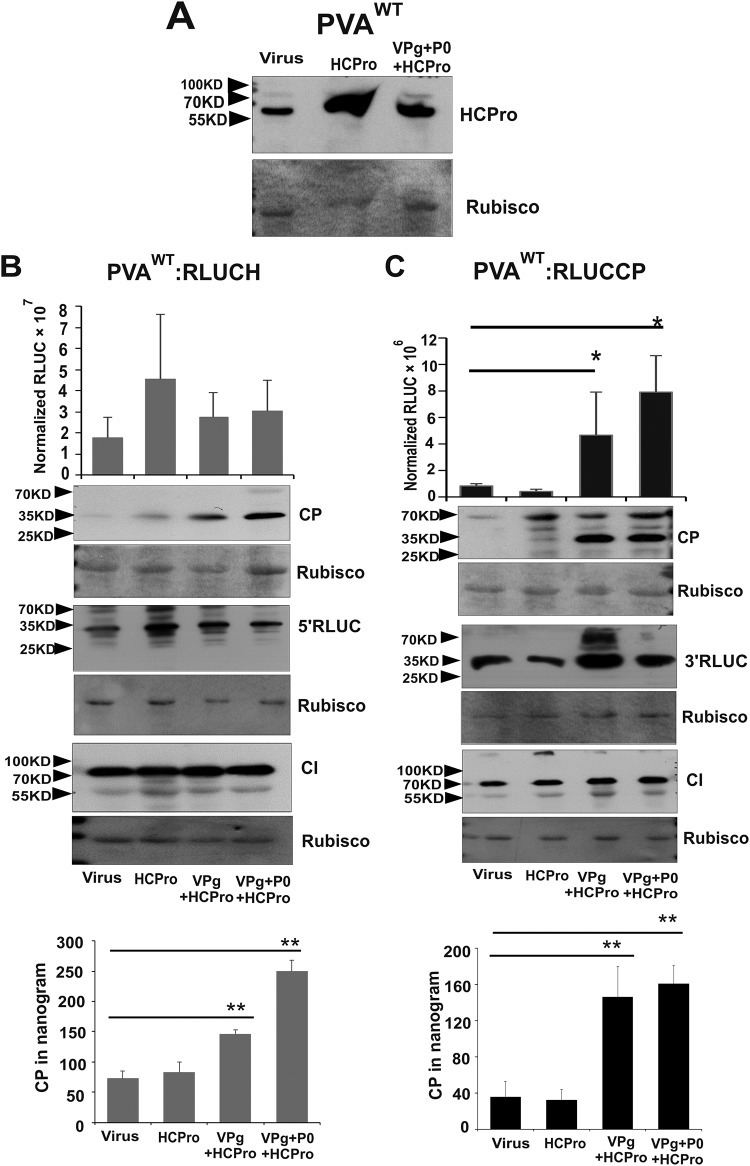
CP accumulation is enhanced by VPg and P0 similarly as 3′ RLUC in the presence of HCPro. Nicotiana benthamiana plants were *Agrobacterium* infiltrated with either PVA^WT^:RLUCH or PVA^WT^:RLUCP alone or together with HCPro, VPg, and P0 expression constructs, as indicated. Plants infiltrated with virus or virus plus HCPro served as controls. (A) Verification of HCPro overexpression in virus-infected plants. (B and C) Accumulation of 5′ RLUC and 3′ RLUC was monitored by activity assays and Western blot analyses, CI and CP were monitored by Western blot analyses, and CP was monitored by ELISAs in PVA^WT^:RLUCH (B)- and PVA^WT^:RLUCCP (C)-infected plants. Rubisco was used as the loading control. The positions of molecular mass markers are shown on the left. Experiments were performed at least in triplicate. Data represent results from one representative experiment, and the bars display means (*n* = 3 or more) ± SD. Student’s *t* test shows which of the samples are significantly different compared to the virus control (*, *P* < 0.05; **, *P* < 0.01).

**FIG 5:**
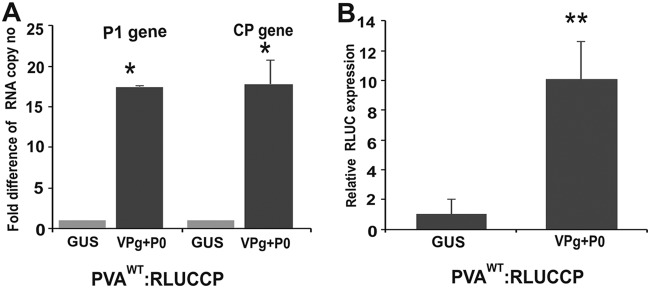
The 5′ and 3′ ends of PVA RNA are present in equal amounts during ectopic VPg+P0 expression. (A) cDNA was synthesized with primers binding to the 5′ P1 and 3′ CP cistrons within PVA^WT^:RLUCCP RNA isolated from infected Nicotiana benthamiana leaf samples (*n* = 3) at 3 dpi. qRT-PCR of the VPg+P0 cDNA gave similar fold differences in RNA copy numbers over the GUS control with both P1 and CP region-specific primers. (B) RLUC assay on the same leaf samples as those for panel A (*n* = 3) reveals a 10-fold difference in 3′ RLUC activity in the presence of VPg+P0 compared to GUS. Experiments were performed in triplicate or more. Data represent results from one representative experiment, and the bars display means ±SD. Student’s *t* test shows which of the samples are significantly different compared to the GUS control (*, *P* < 0.05; **, *P* < 0.01).

To visualize 5′ RLUC and 3′ RLUC on the level of protein accumulation, we subjected the samples collected from PVA^WT^:RLUCH- and PVA^WT^:RLUCCP-infected Nicotiana benthamiana plants expressing GUS, VPg, and P0 in various combinations to Western blot analysis. First, we verified the overexpression of VPg (Fig. 3A). We visualized and compared four proteins, 5′ RLUC, 3′ RLUC, cylindrical inclusion (CI), and CP, by using anti-RLUC, anti-CI, and anti-CP antibodies, respectively. We also quantified CP amounts in these samples by an enzyme-linked immunosorbent assay (ELISA). In PVA^WT^:RLUCH-infected plants, the amounts of 5′ RLUC were equal between all samples (Fig. 3B), whereas 3′ RLUC accumulation was greater in the presence of VPg and VPg+P0 (Fig. 3C). This result is coherent with the RLUC activity assays (Fig. 2A). Levels of CI accumulation were equal for both of the constructs whether coexpressed together with control GUS or with VPg (Fig. 3B and C). Different from 3′ RLUC, neither Western blot analysis nor ELISAs could detect any difference in the CP amounts in spite of VPg and VPg+P0 coexpression (Fig. 3B and C). Since HCPro assists potyviral encapsidation and provides protection against CP degradation (12), we next used HCPro together with VPg and P0 to test CP accumulation (Fig. 4). First, we verified overexpression of HCPro (Fig. 4A). Due to the HCPro addition, 5′ RLUC, CI, and 3′ RLUC were reanalyzed as well. Nicotiana benthamiana plants infiltrated with *Agrobacterium* carrying the PVA^WT^:RLUCCP or PVA^WT^:RLUCH construct alone or together with HCPro were used as controls. Compared to the controls, 3′ RLUC activity was higher (Fig. 4C) and 5′ RLUC activity was not altered significantly (Fig. 4B) in the presence of VPg+HCPro and VPg+P0+HCPro at 3 dpi.

Next, these same samples were subjected to Western blot analysis with anti-CP, anti-RLUC, and anti-CI antibodies. In spite of HCPro addition, 5′ RLUC, 3′ RLUC, and CI accumulated similarly as in the presence of VPg and P0 (compare Fig. 3B and C with Fig. 4B and C). However, an increase in CP accumulation was detected in the presence of VPg+HCPro and VPg+P0+HCPro in both PVA:RLUCH (Fig. 4B)- and PVA:RLUCCP (Fig. 4C)-infected samples compared to the controls. Taken together, these results suggest that coexpression of VPg increases 3′-end CP production similarly to that of 3′ RLUC, but to be stable, CP needs HCPro (compare Fig. 3B and C with Fig. 4B and C). The significantly enhanced CP accumulation achieved during PVA^WT^:RLUCCP and PVA^WT^:RLUCH infection with supplementation with ectopically expressed HCPro, VPg, and P0 could also be verified by an ELISA (Fig. 4B and C, bottom).

### VPg-mediated enhancement in PVA RNA accumulation is equal for both the 5′ and 3′ ends.

No subgenomic RNAs are associated with potyvirus infection. However, the presence of molecules containing the 3′ end of PVA RNA generated by any mechanism could explain boosted protein accumulation from the 3′ end during VPg-mediated translational enhancement. To test if the presence of VPg+P0 induced enhanced accumulation of 3′-end-containing RNA molecules over the 5′-end-containing ones, N. benthamiana leaves were infiltrated with *Agrobacterium* carrying PVA^WT^:RLUCCP and either GUS or VPg+P0 constructs. cDNA was synthesized with primers binding specifically to the P1 cistron at the 5′ end of PVA RNA and to the CP cistron at the 3′ end. The actual copy numbers quantitated by 5′-end-specific qRT-PCR were 1.5- to 4-fold higher than those quantitated by 3′-end-specific qRT-PCR. This difference was derived from both GUS control and VPg+P0 samples, indicating differences in the absolute sensitivities of detection with the chosen gene-specific primers. However, when the 5′- and 3′-end copy numbers of the GUS control were set to a value of 1, the relative level of enhancement in the RNA copy numbers of the VPg+P0 samples was the same for both ends, being approximately 17-fold (Fig. 5A). This indicates that molecules containing the PVA RNA 3′ end were not more abundantly represented in VPg+P0 samples than those containing the 5′ end. In the same samples, the relative enhancement of 3′ RLUC accumulation by VPg+P0 was approximately 10-fold (Fig. 5B). As quantitation was done from total RNA samples, the RNA being measured contains both packaged nontranslatable as well as translatable RNA. Our conclusion is that the enhanced 3′ RLUC or CP accumulation from PVA RNA is not a result of the presence of a disproportionate amount of 3′-end-containing PVA RNA molecules.

### A greater difference is observed in 3′ RLUC and CP than in 5′ RLUC and CI accumulation when their expressions from PVA^WT^ and PVA^ΔGDD^ are compared.

**FIG 6:**
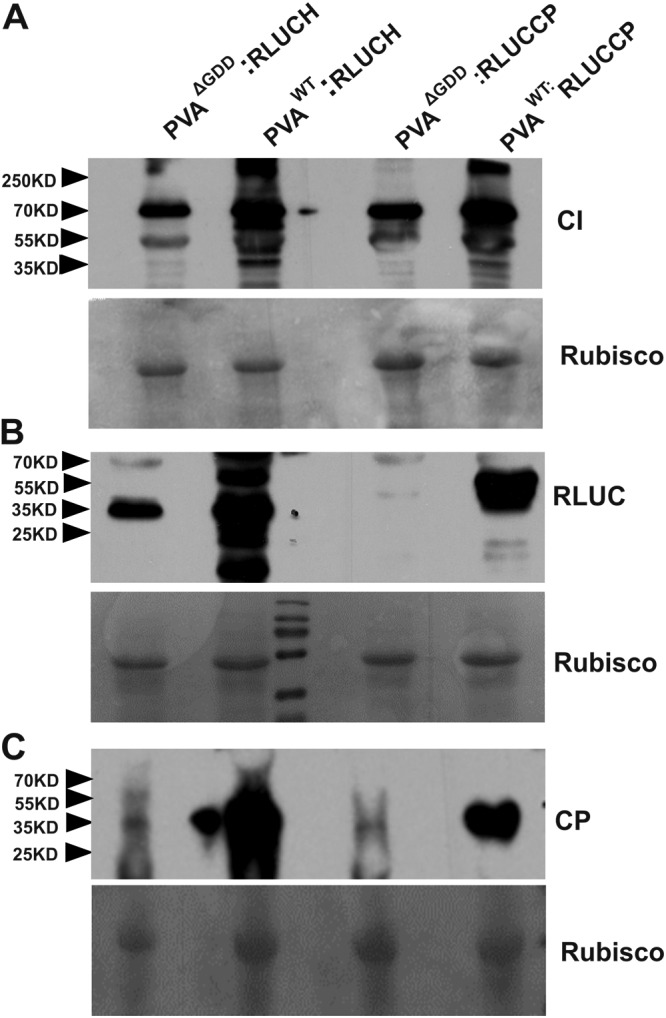
The difference in 3′ RLUC and CP accumulation from PVA^WT^ versus PVA^ΔGDD^ is greater than that in 5′ RLUC and CI accumulation. Nicotiana benthamiana plants were *Agrobacterium* infiltrated with either PVA^WT/ΔGDD^:RLUCH or PVA^WT/ΔGDD^:RLUCP. Samples were collected at 5 dpi, and Western blot analysis was subsequently carried out with anti-CI antibodies (A), anti-RLUC antibodies (B), and anti-CP antibodies (C). Rubisco was used as a loading control in each panel. The positions of molecular mass markers (in kilodaltons) are shown on the left.

The results so far prove that under the ectopic expression of VPg, neither 5′ RLUC nor CI accumulation was affected, whereas viral RNA, 3′ RLUC, and CP accumulation was enhanced. The next important question to address was whether any disproportionate accumulation of PVA proteins could occur during the infection process when no viral or host proteins were overexpressed. We monitored the accumulation of 5′ and 3′ RLUC as well as CI and CP from both the PVA:RLUCCP and PVA:RLUCH constructs at various time points. N. benthamiana plants were *Agrobacterium* infiltrated with PVA^WT^:RLUCCP, PVA^WT^:RLUCH, and their nonreplicating counterparts PVA^ΔGDD^:RLUCCP and PVA^ΔGDD^:RLUCH. We visualized RLUC, CI, and CP accumulation from both the PVA:RLUCCP and PVA:RLUCH constructs by Western blotting (Fig. 6). As can be expected, in general, protein accumulation was greater from the replicating virus than from the nonreplicating virus. The comparison between the replicating and nonreplicating viruses revealed that the difference in 3′ RLUC and CP accumulation levels was much greater than that in CI and 5′ RLUC levels (Fig. 6A to C). In spite of being only a semiquantitative method, the relative amounts of the proteins on the Western blot image revealed a pattern supporting disproportionate protein production from the 3′ end of replicating PVA RNA compared to the translation of the corresponding nonreplicating PVA RNA.

**FIG 7:**
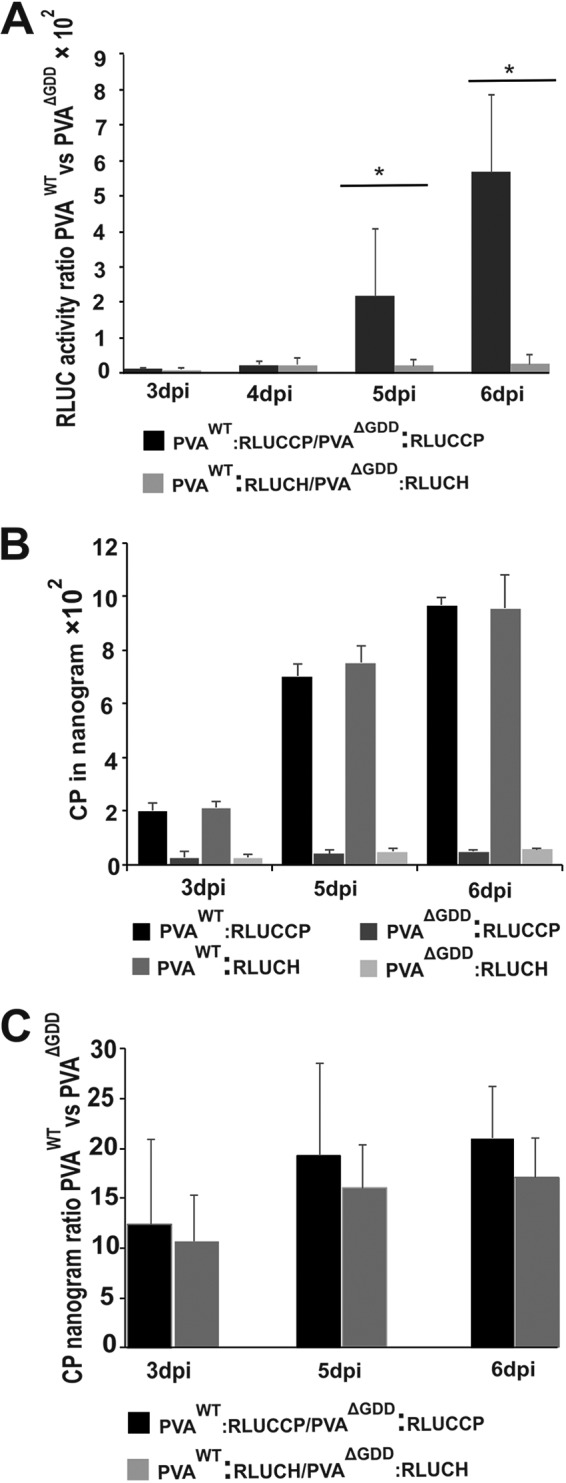
3′ RLUC accumulation accelerates more than that of 5′ RLUC toward the end of PVA^WT^ infection, while CP accumulation is similar from both PVA constructs. *Agrobacterium* cells carrying PVA^WT/ΔGDD^:RLUCCP and PVA^WT/ΔGDD^:RLUCH constructs were infiltrated into Nicotiana benthamiana leaves, and samples were collected between 3 and 6 dpi. (A) Quantitation of RLUC activities. PVA^WT^/PVA^ΔGDD^ activity ratios were calculated from normalized RLUC values, as indicated. Activities from the corresponding PVA^ΔGDD^ RNAs were used as the reference point. (B) CP concentrations determined by an ELISA. (C) Calculation of PVA^WT^ CP/PVA^ΔGDD^ CP ratios based on the ELISA results in panel B. Experiments were performed in triplicate. Data represent results from one representative experiment, and the bars display means (*n* = 3 or more) ± SD. Student’s *t* test revealed significant differences in the RLUC activity ratios between PVA:RLUCCP and PVA:RLUCH samples (*, *P* < 0.05).

Next, we quantitated the 5′- and 3′-end differences in the protein production dynamics from PVA RNA with RLUC activity. For this purpose, N. benthamiana plants were *Agrobacterium* infiltrated with the PVA^WT^:RLUCCP, PVA^ΔGDD^:RLUCCP, PVA^WT^:RLUCH, and PVA^ΔGDD^:RLUCH constructs. Samples were collected at four time points (3 dpi, 4 dpi, 5 dpi, and 6 dpi) to determine the RLUC activities. To have a reference point for PVA^WT^ RLUC activity, we used RLUC activity from PVA^ΔGDD^ RNA as a baseline. The fold differences in PVA^WT^/PVA^ΔGDD^ RLUC activity ratios were calculated for both PVA:RLUCH and PVA:RLUCCP (Fig. 7A). The PVA^WT^:RLUCCP/PVA^ΔGDD^:RLUCCP 3′RLUC activity ratio increased toward the later time points significantly. By 6 dpi, 3′ RLUC activity from the replicating PVA:RLUCCP construct was already hundreds of times higher than that of the nonreplicating PVA:RLUCCP construct. The PVA^WT^:RLUCH/PVA^ΔGDD^:RLUCH ratio showed less enhancement toward the end of infection and was 27-fold at 6 dpi. This result is consistent with the RLUC Western blot image in Fig. 6. We quantitated CP accumulation from PVA^WT^ and PVA^ΔGDD^ for both PVA:RLUCH and PVA:RLUCCP viruses by an ELISA at 3 dpi, 5 dpi, and 6 dpi. CP amounts derived from PVA^WT^:RLUCCP and PVA^WT^:RLUCH were comparable at every time point (Fig. 7B), and the same held true for PVA^ΔGDD^:RLUCCP and PVA^ΔGDD^:RLUCH. We then calculated the ratio of CP accumulation from replicating versus nonreplicating PVA RNA. The CP/CP ratios of PVA^WT^ versus PVA^ΔGDD^ were comparable for both constructs at every time point (Fig. 7C), suggesting that CP production and degradation occur with similar dynamics for both viruses regardless of the position of the *rluc* cistron.

### Viral RNA, 3′ RLUC, and CP accumulate with different dynamics than 5′ RLUC and CI in the course of PVA infection.

**FIG 8:**
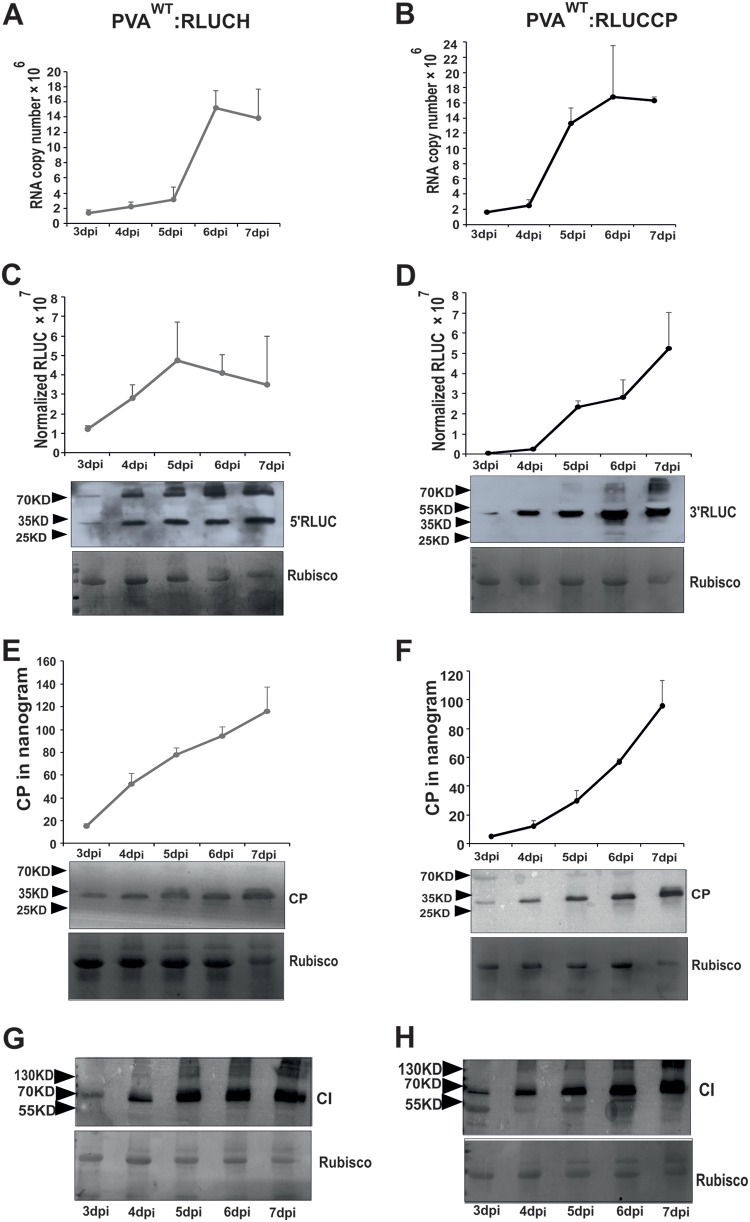
PVA RNA, 3′ RLUC, and CP, unlike 5′ RLUC and CI, accumulate with similar dynamics in the course of PVA^WT^ infection. N. benthamiana plants were infiltrated with *Agrobacterium* carrying PVA^WT^:RLUCH and PVA^WT^:RLUCCP constructs. We monitored the dynamics of viral protein and RNA accumulation between days 3 and 7 postinfection. (A and B) PVA RNA accumulation quantitated by qRT-PCR; (C and D) 5′ and 3′ RLUC activity determination and Western blotting; (E and F) CP determination via an ELISA and Western blotting; (G and H) CI accumulation visualized by Western blotting. Rubisco was used as a loading control in each Western blot. Experiments were repeated four times. Data represent results from one representative experiment, values are means (*n* = 4), and SD are indicated.

In the next set of four independent experiments, we monitored CI, CP, RLUC, and RNA accumulation from both PVA^WT^ viruses in the course of infection. We *Agrobacterium* infiltrated N. benthamiana plants with PVA^WT^:RLUCCP and PVA^WT^:RLUCH constructs and quantitated RLUC activities as well as CP and RNA amounts daily between 3 and 7 dpi (Fig. 8). qRT-PCR results indicate that the dynamics of RNA accumulation from both PVA^WT^:RLUCH (Fig. 8A) and PVA^WT^:RLUCP (Fig. 8B) were similar, with the relative fold increase in RNA copy numbers being approximately 10 between days 3 and 7. The graphs show that PVA RNA accumulation reached its top value by day 6 in both cases.

The relative 5′ RLUC and 3′ RLUC activities were plotted against the time of infection (Fig. 8C and D). PVA infection initiated via *Agrobacterium* infiltration at an optical density (OD) of 0.05 has not yet reached all of the cells in the infected leaf area by day 4 (23), and therefore, we interpret that the spread of the virus increases the number of infected cells up to day 5. The 5′ RLUC activity did not increase anymore after 5 dpi, whereas the 3′ RLUC activity continued to rise up to day 7. The maximum fold increases in RLUC activities between days 3 and 7 are 2.8 for 5′ RLUC and 112 for 3′ RLUC in this representative experiment. The great fold difference in 3′ RLUC accumulation is mainly due to its very low activity in the beginning of infection. These data suggest that 5′ RLUC and 3′ RLUC activities change with different dynamics in the course of infection. While 5′ RLUC accumulation was already pronounced by day 2 and reached its maximum by day 5 of infection, the negligible initial amount of 3′ RLUC continued to increase throughout the 7 days of the experiment. Although Western blotting is a semiquantitative analysis, a similar trend in RLUC protein and activity accumulation can be observed (Fig. 8C and D).

In the presented experiment, the CP concentration produced by PVA^WT^:RLUCH infection was 1.2-fold higher than that produced by PVA^WT^:RLUCCP at day 7 (Fig. 8E and F), indicating that both viruses produced fairly similar CP titers. CP accumulation increased steadily throughout the experiment from both viruses and reached its top value by day 7. An increasing trend in CP accumulation can be observed from the Western blot analyses as well (Fig. 8E and F). When Western blot analysis was carried out with anti-CI antibodies, CI accumulation seemed to be nearly steady from day 5 on. Taken together, data from this experiment suggest that while the viral RNA amount increased up to day 6 and the 3′ RLUC activity and CP amount increased up to day 7, 5′ RLUC and CI accumulation reached a plateau at day 5 in the infected leaves. Therefore, together with the other results of this study, this result also supports the view that accumulation of 5′ RLUC and CI follows a different pattern than that of PVA RNA, 3′ RLUC, and CP.

**FIG 9:**
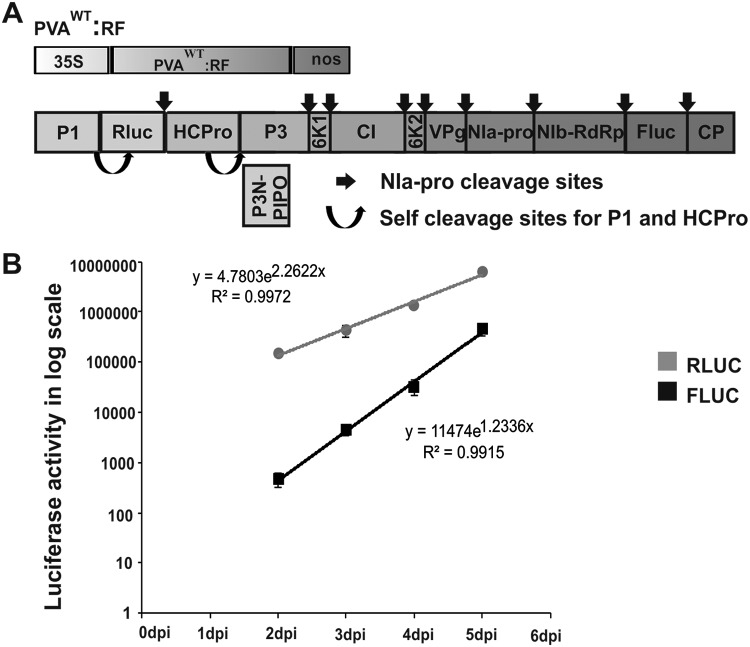
3′ FLUC accumulates at a higher rate than 5′ RLUC toward the end of PVA^WT^ infection when expressed from the same PVA RNA. (A) Schematic representation of PVA^WT^:RF, having the *rluc* gene at the 5′ end and the *fluc* gene at the 3′ end. The PVA^WT^:RF construct allows expression of 5′ RLUC from a location between the P1 and HCPro cistrons and of 3′ FLUC from a location between NIb and CP. (B) N. benthamiana plants were infiltrated with *Agrobacterium* carrying PVA^WT^:RF, and samples were collected daily between 2 and 5 dpi. RLUC and FLUC concentrations were calculated and are plotted as a function of time. Experiments were repeated three times. Data represent results from one representative experiment, values are means (*n* = 4), and SD are indicated.

Furthermore, we wanted to verify that a similar difference in 5′- and 3′-end gene expression could be observed when the viral proteins are coming from the same PVA genome. This was studied with a construct carrying the *rluc* gene (5′ RLUC) between the P1 and HCPro cistrons similarly as in PVA^WT^:RLUCH and the *fluc* gene (3′ firefly luciferase [FLUC]) between NIb and CP of a PVA icDNA similarly as the *rluc* gene in PVA^WT^:RLUCCP (Fig. 1B). This PVA icDNA construct was named PVA^WT^:RF (Fig. 9A). We *Agrobacterium* infiltrated N. benthamiana plants carrying the PVA^WT^:RF construct at an OD of 0.01 and measured both RLUC and FLUC activities in the infected leaf samples daily from 2 to 5 dpi. The activities were plotted as a function of time in a graph (Fig. 9B). In line with our above-described results, 5′ RLUC accumulation, which was already pronounced at 2 dpi, increased at a moderate rate until 5 dpi. 3′ FLUC accumulation, which was minuscule at 2 dpi, increased at a higher rate than 5′ RLUC accumulation until 5 dpi. The maximum fold increases in LUC activities between days 2 and 5 are 42 for 5′ RLUC and 960 for 3′ FLUC in this representative experiment. Thus, on a relative scale, 3′ FLUC activity increases 23 times more than 5′ RLUC activity during days 2 to 5 of infection. Comprehensively, the results of this experiment are in line with our suggestion of nonsimultaneous accumulation of viral proteins derived from either the 5′ or 3′ end of the PVA genome in the course of infection.

## DISCUSSION

In the present study, we examined the accumulation of PVA proteins expressed from two PVA icDNA constructs, with the difference being the position of the inserted *rluc* cistron. Viral protein amounts were quantitated in the first set of experiments in PVA:RLUCH- and PVA:RLUCCP-infected N. benthamiana leaves overexpressing VPg and auxiliary proteins and in the second set in infection where no viral or host proteins were overexpressed. As the main result supported by all experiments, we found that PVA CP, translated from the 3′-most cistron, and 3′ RLUC expressed from a position in front of the CP cistron accumulate with different dynamics than CI, translated from the middle of the viral RNA, and 5′ RLUC from a cistron located in front of the HCPro-encoding sequence. The different rates of 5′ RLUC and 3′ FLUC accumulation from a single PVA RNA molecule further supported the suggestion that protein accumulation from the 3′end of PVA RNA follows different dynamics than that from the rest of the genome. Based on the findings of this study, we discuss the possibility that these observations connect with putative mechanisms by which potyvirus infection limits CP accumulation during early infection and that of replication proteins late in infection.

Accumulation of a particular protein in the cell is a sum of its production and degradation rates. Even if produced with an equal rate from a polyprotein, the steady-state amounts of different viral proteins may vary substantially because of different turnover rates. There is a 1:1 linear correlation between the activity of RLUC and its concentration, respectively (https://fi.promega.com/resources/product-guides-and-selectors/protocols-and-applications-guide/bioluminescent-reporters/). The half-lives of RLUC and FLUC are short, 4.5 and 3 h, respectively (25), and due to their short half-lives, they can be used to report dynamic gene expression responses. We see from Fig. 1 that 3′ RLUC was fully processed, while partial 5′ RLUC processing was observed. C-terminal fusions to RLUC may affect its activity, as was found previously (26) with dual-luciferase reporter fusions. Western blot analyses in this study reveal that although a significant change in the 3′ RLUC activity occurred, there was no significant change in its protein-processing pattern. Therefore, we interpret that proteolytic processing did not affect the changes observed in either 5′ RLUC or 3′ RLUC activities. Changes in 5′ RLUC or 3′ RLUC stabilities could affect the results. This would require destabilization of 5′ RLUC and stabilization of 3′ RLUC in the course of infection or upon VPg expression, but a change to both directions is doubtful. The third alternative is that the RLUC activities reflect the dynamic changes in RLUC production. This could be achieved by an alternative translational strategy or by regulation of translation via RNA binding proteins on polysomes.

CI forms pinwheel-shaped cytoplasmic inclusion bodies during potyvirus infection (27). They form early in infection, when active genome translation and replication are still ongoing (28), as a coordinated action of CI and P3N-PIPO (29). Association of the conical CI structures with plasmodesmata is essential for potyviral cell-to-cell movement (29). This self-assembly property likely increases the half-life of CI. Therefore, the amount of CI may reflect the accumulation of CI over a longer period than just the rate of production.

Regulation of PVA CP stability is complex. PVA CP phosphorylation and the host chaperons HSP70/CPIP have the capacity to direct PVA CP toward proteasomal degradation, and this is essential for viral replication and translation (9). Since the flexuous filamentous virions of potyviruses are composed of approximately 2,000 copies of CP, virion assembly obviously requires a large amount of CP (30). HCPro stabilizes CP and enhances potyviral particle formation (12). In addition, P3 contributes to virion formation, and the virus to be encapsidated needs to be able to replicate (13). We previously proposed that the gradual increase of CP toward the later stages of infection eventually overpowers HSP70/HSP40 regulation (5, 11). Cessation of CP degradation together with promotion of virion formation by HCPro could as such ensure enough CP for particle formation. Particle formation also stabilizes viral RNA and CP, which could explain their continuous accumulation throughout the infection process (Fig. 8). Although particle formation contributes to CP and PVA RNA amounts by stabilizing them, it cannot explain the results of this study sufficiently. What is puzzling is that while 5′ RLUC and CI accumulation remains at a nearly constant level, there is a simultaneous increase in 3′ RLUC and 3′ FLUC accumulation.

Of interest is that 3′ RLUC expression is substantially upregulated by VPg from both replicating and nonreplicating RNAs, the latter of which is not encapsidated according to current knowledge (12, 13). Since we gained the initial evidence for the enhanced accumulation of proteins from the 3′ end by quantitating virus-derived reporter gene expression, we were concerned about possible artifacts due to the nonnatural reporter gene sequence in the PVA genome. We previously demonstrated that overexpression of VPg concomitantly enhances 3′ RLUC production and stabilizes PVA RNA (14). Importantly, stabilization of PVA RNA was observed here regardless of the position of *rluc* in the viral genome, objecting to the role of the 3′ *rluc* sequence in this phenomenon. In addition, a VPg-mediated boost in CP expression was observed in the presence of overexpressed HCPro irrespective of the position of *rluc*. Therefore, it is possible that in PVA:RLUCCP RNA, the 3′ *rluc* cistron, which is in front of the CP cistron, is under the same regulation as CP. As VPg upregulates the expression of CP independently of the *rluc* sequence at the 3′ end of viral RNA, it can represent a mechanism utilized during natural PVA infection.

While the expression of viral proteins is greater from replicating than from nonreplicating viral RNA in general (Fig. 6), there is a distinct speedup of 3′ RLUC and CP expression on top of that. On the contrary, enhancement of 3′-end expression and RNA accumulation in a VPg-overexpressing background occurs for both replicating and nonreplicating viral RNAs (Fig. 2). This raises the question of whether the mechanism of upregulation is the same in the presence of overexpressed VPg and during the late stages of PVA infection. Assuming that the mechanism is the same in both cases, at least one explanation can be envisioned: it is possible that translation of the nonreplicating PVA RNA does not provide a sufficient source of VPg, as our previous study demonstrated that VPg-mediated enhancement is concentration dependent (14).

Multiple translational strategies are exploited for the dynamic regulation of viral protein production (reviewed in reference 31). Such regulation is pivotal to retain correct amounts of each viral protein to serve infection. The mechanisms utilized to initiate viral translation are via scanning, shunting, and internal ribosome entry sites (IRESs) (reviewed in reference 32). Interestingly, there are three distinct translation initiation mechanisms described for human immune deficiency virus structural protein Gag production, as explained previously (33–35). A recent study on the Gag-IRES molecular mechanism gave evidence for multiple translation events during infection (36). This example shows that the structural proteins of viruses can in some virus groups be produced by multiple mechanisms even if they use polyprotein production as the translational strategy. In the light of the above-mentioned example, the existence of an alternative translational mechanism for potyviral CP production as an explanation for our observations cannot be ruled out. Another possibility is that mechanisms to inhibit CP production at the beginning of infection and replication proteins late in infection by RNA binding proteins exist.

We know that PVA VPg, the 5′ UTR of PVA RNA, and other viral and host proteins coregulate 3′ RLUC expression together with VPg (14–16). The coregulators identified so far are HCPro, ribosomal protein P0, eIF4E/(iso)4E, and VCS. It is intriguing that all of these proteins are constituents of the RNA granules induced by HCPro during PVA infection (16). Turnip mosaic virus (TuMV) VPg was recently found to resist autophagy-mediated degradation of TuMV HCPro-induced RNA granules (37). We suggested previously (16) that PVA-induced granules are a consequence of HCPro-mediated RNA silencing suppression required to protect viral RNA. In addition to its role in directing viral RNA toward translation, it appears that VPg has a role in blocking the route of these granules to degradation by autophagy (37). VPg’s role in these functions could therefore contribute to upregulated PVA RNA, CP, and 3′ RLUC accumulation. It has been estimated that TuMV enters a new cell layer every 3 h in N. benthamiana (38). Pronounced CI and 5′ RLUC expression in early PVA infection could reflect the requirement of the replication proteins at the infection front. In spite of the fast cell-to-cell spread of infection, viral gene expression and particle accumulation still continue in the infected cells. While PVA movement is not affected in P0-, VCS-, and eIF4E/eIF(iso)4E-silenced backgrounds, there are great reductions in 3′ RLUC and 3′ green fluorescent protein (GFP) production and in viral RNA and virion accumulation (15, 16). We have shown that binding of CP to PVA RNA blocks viral translation (9, 10) and proposed that this block is required to allow time for the formation of the replication complex. As the binding of CP occurs at the 3′ end, translation may continue from the 5′ end, which could explain the stronger production of the 5′-end than of the 3′-end proteins in early infection. In addition, we propose that later in infection, VPg, together with HCPro, P0, VCS, and eIF(iso)4E, contributes specifically to CP production, stabilization, and particle formation.

## MATERIALS AND METHODS

### Gene constructs.

Viral constructs used in this study are based on the full-length cDNA of PVA strain B11 (GenBank accession number AJ296311) (39). The 35S-PVA^wt^::*rluc*^int^-nos construct (23) carries the *Renilla* luciferase reporter gene (*rluc*) and the first intron of ribulose-1,5-bisphosphate carboxylase/oxygenase (Rubisco) (RBC-1I) within it to prevent bacterial gene expression. We have typically called the virus derived from this construct PVA^WT^ in our previous publications, but for the purposes of the present study, we renamed it PVA^WT^:RLUCCP to emphasize the 3′-end position of the *rluc* cistron. Its replication-deficient variant was named PVA^ΔGDD^:RLUCCP. Another 35S-PVA^wt^::*rluc*^int^-nos construct, in which the *rluc* fragment was cloned into a cloning site preceding the HCPro cistron, was described previously (24). RLUC produced from this site is surrounded by two functional protease sites to confirm the cleavage of RLUC from the rest of the genome. The virus derived from this construct was called PVA^OLO^ previously (24), but in the present study, it was named PVA^WT^:RLUCH, and its replication-deficient variant was named PVA^ΔGDD^:RLUCH, to emphasize the 5′ position of the *rluc* cistron. The protein expression constructs 35S-*fluc-nos* (23), 35S-VPg-*nos*, 35S-*uidA-nos* (14), 35S-P0-*nos* (15), and 35S-HCPro-*nos* (16) were all previously reported.

### Plants.

Nicotiana benthamiana plants were used in this study. Plants were grown in a greenhouse at 22°C for 18 h in light and at 18°C for 6 h in darkness. Plants were infected at the 4- to 6-leaf stage as described previously (23).

### *Agrobacterium* infiltration and sample collection.

For *Agrobacterium* infiltration, we used Agrobacterium tumefaciens strain C58C1 (40) containing the helper plasmid pGV2260 for *vir* gene expression and carrying the required viral and protein expression constructs. The cells were grown overnight in Luria-Bertani (LB) medium at 28°C in the presence of adequate antibiotics. The cells were harvested by centrifugation at 3,000 × *g* for 5 min and washed once in double-distilled water, after which they were suspended in induction buffer (10 mM morpholineethanesulfonic acid [MES] [pH 6.3], 10 mM MgCl_2_, and 150 μM acetosyringone). The final *Agrobacterium* densities were adjusted, and the cells were incubated for 2 to 3 h in induction buffer at room temperature (RT) prior to infiltration. For *Agrobacterium* carrying PVA constructs, an optical density at 600 nm (OD_600_) of 0.05 was used unless otherwise stated, and for *Agrobacterium* carrying protein expression constructs, an OD_600_ of 0.5 was used. As an internal control for transformation efficiency, we used firefly luciferase (FLUC), which was infiltrated at an OD_600_ of 0.005. *Agrobacterium* cultures were mixed with each other at the required ratios to form the ﬁnal inﬁltration culture, which was then delivered to the abaxial side of N. benthamiana leaves with a syringe. Sampling was done by cutting 5- to 10-mm leaf discs with a cork borer to form a circle surrounding the infiltrated region at many different time points, as explained in Results. The collected samples, each consisting of four leaf discs, were frozen immediately in liquid nitrogen. Each experiment was performed at least twice with a minimum of three biological replicates.

### Quantification of viral gene expression by an RLUC assay.

We quantitated viral gene expression by an RLUC assay essentially as described previously (8, 14). The samples were prepared according to the manufacturer’s instructions for the dual-luciferase kit (Promega), and activities were measured using a Luminoscan TL Plus instrument (Thermo Labsystems). RLUC normalization was done with the following formula: normalized RLUC activity = (average FLUC activity/FLUC activity per sample) × RLUC activity per sample. Average normalized RLUC values and their standard deviations (SD) were calculated. Student’s *t* test was employed to calculate the significance of the differences between the experimental and control samples.

### Reverse transcription-quantitative PCR.

For reverse transcription-quantitative PCR (qRT-PCR), N. benthamiana leaf discs were collected at different time points as indicated in Results. Each sample set represents a pooled sample from four biological replicates. RNA isolation was carried out with an RNeasy plant minikit (Qiagen) according to the manufacturer’s protocol. One microgram of total RNA from each sample was treated with RNase-free DNase (Thermo Scientific). First-strand cDNA synthesis was performed by using a RevertAid H Minus first-strand cDNA synthesis kit (Thermo Scientific) according to the user protocol. Quantitative PCR (qPCR) was performed in 96-well plates in a 10-μl reaction mixture volume, using a CFX96 Touch real-time PCR system (Bio-Rad). Three technical replicates were conducted for each cDNA. Each 10-μl PCR mix contained 5 μl Maxima SYBR green qPCR master mix (Thermo Scientific), 0.5μM each forward and reverse primers, 1 μl cDNA, and 3 μl nuclease-free water. The following primer pairs were used for amplification of P1 and CP cistron regions: P1qPCRF′ (5′-CTGGTGACTGGGCAAAGAAT-3′)/P1qPCRR′ (5′-TGAGAGCATGGTGGACTCTG-3′) and CPqPCRF′ (5′-CATGCCCAGGTATGGTCTTC-3′)/CPqPCRF′ (5′-ATCGGAGTGGTTGCAGTGAT-3′). The protein phosphatase 2A (PP2A) gene, a housekeeping gene, was used as a reference gene. PP2A was amplified with the primers as described previously (41). The amplification parameters for qPCR were 3 min of initial denaturation at 95°C, followed by 39 cycles of 10 s of denaturation at 95°C, 30 s of annealing 55°C, and 30 s of synthesis at 72°C. A melting curve was generated by heating from 60°C to 95°C in increments of 0.5°C/s. The following controls were included: a template replaced by nuclease-free distilled water (dH_2_O) as a nontemplate control and RT reaction mixtures lacking the reverse transcriptase as non-RT controls. A serial dilution of PVA icDNA was used, and the quantification cycle (*C_q_*) values were plotted against the values for the input cDNA to construct the standard curve.

### SDS-PAGE and Western blot analysis.

Samples for SDS-PAGE and Western blot analysis were collected from 4 to 5 similarly treated plants and contained six leaf discs each. They were frozen immediately in liquid nitrogen, ground to a fine powder, suspended in SDS-containing protein extraction buffer (25 mM Tris-HCl, 2% SDS), and heated with Laemmli sample buffer containing β-mercaptoethanol at 95°C for 5 min. Proteins were separated on 12% SDS-polyacrylamide gels. After the run, proteins were blotted onto polyvinylidene difluoride (PVDF) membranes (Immobilon-P; Millipore). The membranes were blocked in 3% bovine serum albumin (BSA), dissolved in Tris-buffered saline (TBS) (50 mM Tris-HCl [pH 7.4], 150 mM NaCl) containing 0.1% Tween 20, and probed with anti-RLUC monoclonal antibody (MAb) (1:1,0000) (clone 5B11.2; Millipore), affinity-purified anti-VPg IgGs (1:10,000), anti HCPro IgGs (1:10,000), or anti-CP antiserum (1:10,000). Detection with horseradish peroxidase (HRP)-conjugated secondary antibodies (1:15,000; Promega) was carried out using the Immobilon Western chemiluminescent HRP substrate (Millipore). A Rubisco band either stained with Ponceau S or detected by Western blotting using Rubisco polyclonal anti-rabbit antibody (Agrisera, Sweden) was used as a loading control.

### ELISA.

For ELISAs, 6 leaf discs were collected from individual plants at different time points, frozen immediately in liquid nitrogen, and ground to a fine powder. Five hundred microliters of sample extraction buffer (washing buffer, 8 M polyvinylpyrrolidone [PVP], 0.2% BSA) was added to each sample and suspended. Four to five biological replicates were analyzed. The wells of a flat-bottom 96-well plate (Costar 3590) were coated with primary antibody (PVA mix MAb; Science and Advice for Scottish Agriculture [SASA], Edinburgh, UK) diluted 1:1,000 in coating buffer (15 mM Na_2_CO_3_, 34 mM NaHCO_3_ [pH 9.6]) by incubating the plates for 3 h at 37°C. Plates were washed a further 3 to 4 times with wash buffer (1.4 mM KH_2_PO_4_, 8 mM Na_2_HPO_4_, 136 mM NaCl, 2.6 mM KCl [pH 7.4], 0.05% Tween 20). One hundred microliters of the sample was loaded into each well. A concentration gradient of purified PVA particles dissolved in mock-inoculated plant sap was made and loaded similarly. A mock-inoculated plant sample was used as a blank. The plates were kept at 4°C overnight, after which the wells were washed again similarly as described above and incubated for 3 h with enzyme-conjugated secondary antibody (1:4,000) (PVA58/0 alkaline phosphatase [AP]; SASA) in extraction buffer. The wells were washed again 3 to 4 times with wash buffer and tap dried. A 100-μl solution of *p*-nitrophenyl phosphate (phosphatase AP; Sigma), dissolved in substrate buffer (9.7% diethanolamine [pH 9.8]), was added to each well. The color reaction was quantitated spectrophotometrically at a 405-nm wavelength in a Tecan infinite m200 instrument. Measurements were carried out 2 to 3 times in 40- to 60-min intervals after the color started to develop. A standard curve was generated from the concentration gradient of PVA particles, and it was used to determine PVA CP concentrations in experimental samples.
